# The association between HbA1c/HDL-C and the incidence of cardiometabolic multimorbidity in middle-aged and elderly adults: Results from the China Health and Retirement Longitudinal Study

**DOI:** 10.1371/journal.pone.0332376

**Published:** 2025-10-23

**Authors:** Yuan Zeng, Lin Lu, Wenwu Zheng, Gong Chen

**Affiliations:** 1 Department of Cardiology, The Affiliated Hospital, Southwest Medical University, Luzhou, Sichuan Province, China; 2 Institute of Cardiovascular Research, The Southwest Medical University, Luzhou, Sichuan Province, China; 3 Department of Cardiology, The First People’s Hospital of Yuexi County, Liangshan Prefecture, Sichuan Province, China; Bushehr University of Medical Sciences, IRAN, ISLAMIC REPUBLIC OF

## Abstract

**Background:**

Cardiometabolic multimorbidity (CMM) refers to a cluster of metabolic and cardiovascular diseases, including stroke, diabetes, and cardiovascular diseases. Glycated hemoglobin (HbA1c) and high-density lipoprotein cholesterol (HDL-C) are widely used to assess glucose metabolism and lipid metabolism disorders, respectively, given their close association with the onset of diabetes and cardiovascular diseases. This study aimed to investigate the association between the HbA1c/HDL-C ratio and the incidence of newly diagnosed CMM in individuals aged 45 and older, using data from the China Health and Retirement Longitudinal Study (CHARLS) database.

**Method:**

Our study utilized data from the CHARLS database from 2011 to 2018. A multivariate logistic regression model was established to determine the association between the cumulative average HbA1c/HDL-C (2011–2015) and the incidence of newly diagnosed CMM (2015–2018). Besides, a multivariable-adjusted restricted cubic spline (RCS) curve, stratified analysis, and interaction tests were conducted to evaluate the relationship between HbA1c/HDL-C and CMM.

**Results:**

Our longitudinal analysis included 4,225 participants, with 716 new cases of CMM identified over a 7-year follow-up period. A positive association was observed between the HbA1c/HDL-C ratio and the incidence of CMM. In the fully adjusted model, the highest quartile of HbA1c/HDL-C (Q4) exhibited the highest risk of CMM (OR = 1. 08, 95% CI: 1. 04–1. 12). The association between the HbA1c/HDL-C ratio and CMM incidence was consistent in all subgroups except for education level. Stratified analysis showed no significant interaction between the HbA1c/HDL-C ratio and factors such as age, gender, marital status, education level, residence, smoking status, drinking status, and body mass index (BMI).

**Conclusion:**

A significant linear relationship exists between the HbA1c/HDL-C ratio and the risk of CMM. Accordingly, the HbA1c/HDL-C ratio may be an independent risk factor for CMM. Among middle-aged and older adults, monitoring and managing HbA1c/HDL-C levels may aid in identifying individuals at high risk of developing CMM.

## 1. Introduction

As the global population ages, multimorbidity is emerging as a significant public health challenge and a key focus in global health research. Multimorbidity refers to the simultaneous presence of multiple diseases in an individual due to similar pathogenesis or shared risk factors [1,2]. Despite the complex underlying mechanisms of multimorbidity development, and a limited understanding of how to prevent it, population-level interventions show promise [3,4]. Among the various patterns of multimorbidity, cardiometabolic multimorbidity (CMM) is one of the most studied [5]. CMM refers to the presence of at least two cardiovascular-metabolic diseases simultaneously, such as diabetes, stroke, and heart disease [6]. Any combination of these conditions is associated with a significantly increased risk of mortality, and patients with CMM often experience a much lower life expectancy [7]. Therefore, identifying high-risk populations and implementing effective management and treatment strategies are crucial steps for reducing the incidence of CMM and improving the overall health of middle-aged and older adults.

Glycated hemoglobin (HbA1c) and high-density lipoprotein cholesterol (HDL-C) are widely used biomarkers for evaluating glucose and lipid metabolism disorders and are closely related to the onset of diabetes and cardiovascular diseases. Insulin resistance is the core mechanism underlying the imbalance of glucose and lipid metabolism. When the body’s sensitivity to insulin decreases, it not only leads to elevated blood glucose levels but also triggers lipid metabolism disorders [8]. While HbA1c provides an indicator of long-term glycemic control, HDL-C represents the body’s anti-atherosclerotic capacity. The ratio of HbA1c/HDL-C can thus comprehensively assess the imbalance of glucose and lipid metabolism. Previous studies have reported that HbA1c/HDL-C is associated with carotid atherosclerosis, stroke, and metabolic diseases [9–11]. However, little is known about the contribution of HbA1c/HDL-C to CMM. Therefore, based on data from the China Health and Retirement Longitudinal Study (CHARLS) database from 2011 to 2018, this study aimed to explore the association between HbA1c/HDL-C and CMM in middle-aged and elderly individuals in China, providing new evidence for the prevention and control of CMM.

## 2. Method

### 2.1. Study population

**Fig 1:**
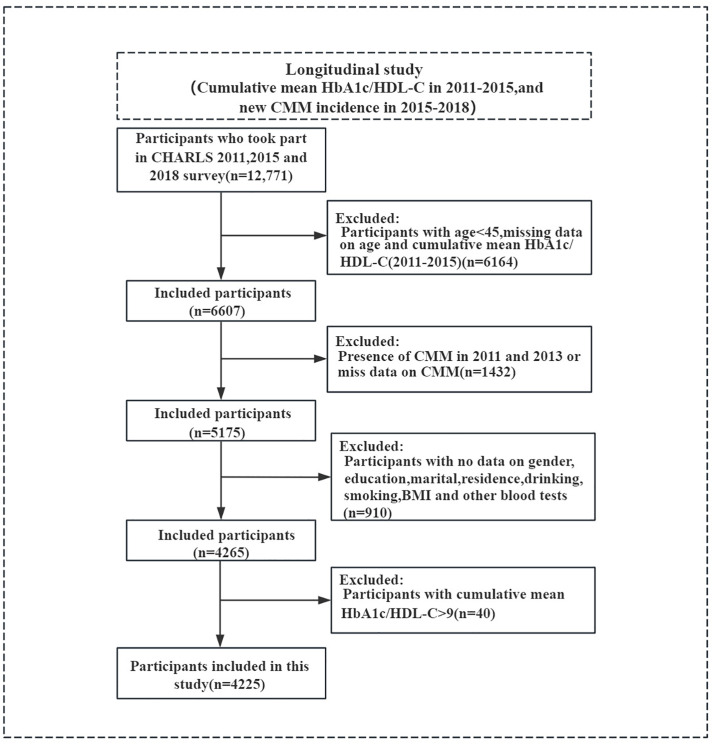
Flowchart of patient selection.

The China Health and Retirement Longitudinal Study is a nationally representative longitudinal survey conducted in China. This study utilized data from CHARLS, a nationally representative survey initiated in 2011 to prospectively collect health data from the Chinese population. Follow-up surveys are conducted biennially, with four waves of national data released to date: 2011, 2013, 2015, and 2018 (data available at http://charls.pku.edu.cn/en) [12]. Ethical approval for CHARLS was obtained from the Peking University Ethics Review Committee (IRB 00001052–11015), and all participants provided informed consent. All procedures involving human subjects adhered to the ethical standards of the institution and the national research committee, as well as the Helsinki Declaration and its subsequent amendments or similar ethical guidelines. As a nationwide survey focused on the health status and influencing factors of the middle-aged and elderly population, CHARLS includes comprehensive data on basic information, family structure, physical health, healthcare and insurance, work and pensions, income and expenditure, housing, and laboratory tests for participants aged 45 and above from 28 provinces. Our study utilized data from the CHARLS database, spanning from 2011 to 2018, a nationally representative longitudinal survey in China that employs a multistage stratified probability sampling method with probability proportional to size (S1 File). Participants included were aged 45 and above who fully participated in the 2011, 2013, 2015, and 2018 survey waves and had complete information on HbA1c, HDL-C, and CMM history. The following exclusion criteria were implemented based on the research objectives: (1) Incomplete information on CMM or CMM incidence in 2011 and 2015; (2) Missing HbA1c/HDL-C data in 2011 and 2015; (3) Extreme values for cumulative average HbA1c/HDL-C (> 9). Additional exclusions were made for missing data on the following key covariates: (1) Age under 45 years; (2) Missing data on gender, marital status, education level, residence, smoking status, drinking status, blood urea nitrogen (BUN), fasting blood glucose (FBG), serum creatinine (Cr), total cholesterol (TC), triglycerides (TG), HDL-C, C-reactive protein (CRP), hemoglobin (Hb), HbA1c, uric acid (UA), and BMI. A total of 4225 eligible participants were finally included (Fig. 1).

### 2.2. Evaluation of HbA1c/HDL-C

After fasting overnight for at least 8 hours, professional staff collected three tubes of venous blood from participants and measured the parameters according to standard procedures. HDL-C was measured using the enzyme colorimetric method for lipid parameters. The HbA1c was determined using the compensated Jaffe creatinine method and the borate affinity high-performance liquid chromatography method. For the present study, HbA1c and HDL-C measurements were obtained from two time points: baseline in 2011 and follow-up in 2015. The cumulative average HbA1c/HDL-C was calculated using the formula: (HbA1c/HDL-C of 2011 + HbA1c/HDL-C of 2015)/ 2. Besides, the continuous exposure variable HbA1c/HDL-C was divided into quartiles for further analysis.

### 2.3. Evaluation of CMM

CMM refers to the simultaneous presence of at least two cardiometabolic diseases (CMD), typically including diabetes, heart disease, and stroke [6]. Diabetes was defined according to the 2005 American Diabetes Association criteria, which include: HbA1c ≥ 6. 5%; FBG ≥ 7 mmol/L; a random blood glucose level ≥11.1 mmol/L; or a self-reported diagnosis (“Have you been diagnosed with diabetes or hyperglycemia by a doctor?”) [13]. Participants diagnosed with coronary heart disease or stroke were primarily identified based on self-reports collected during baseline and follow-up surveys (“Have you been diagnosed with heart disease, coronary artery disease, angina, congestive heart failure, or other heart problems by a doctor?” and “Have you been diagnosed with a stroke by a doctor?”). Participants presenting with a simultaneous diagnosis of two or more of the following conditions: heart disease, stroke, diabetes, or hyperglycemia, were classified as CMM patients [12].

### 2.4. Other variables

In the self-reported questionnaires, data were adjusted based on sociodemographic characteristics, health-related behaviors, anthropometric measurements, and medication use. Sociodemographic variables included: age, gender, education level (“elementary school or below”, “high school”, and “college or above”), residence (“urban/town” and “rural”), and marital status (“married” and “never married/separated/widowed”). Health-related behaviors included: smoking status (“non-smoker”, “former smoker”, and “current smoker”), and drinking status (“never”, “less than once a month”, and “more than once a month”) [14,15]. Anthropometric measurements were taken with participants wearing light clothing and no shoes. Weight was measured using a digital weighing scale (Omron Corporation, HN-286) with an error margin of 0.1 kg, and height was measured using a stadiometer (Seca Corporation, 213) with an error margin of 0.1 cm. BMI (kg/m²) was calculated as weight/height². After overnight fasting, professional staff collected three tubes of venous blood from participants and measured the parameters according to standard procedures. TC, TG, HDL-C, Low-density lipoprotein cholesterol (LDL-C), FBG, CRP, Hb, BUN, Cr, and UA were measured using the enzyme colorimetric method, compensated Jaffe creatinine method, and borate affinity high-performance liquid chromatography method. In addition, the use of antihypertensive, antidiabetic, and antidyslipidemic medications was considered during the analysis.

### 2.5. Missing data handling

The extent of missing data in this study is detailed in S1 Table in S2 File. We adopted a complete case analysis in this study, including only participants with complete data for all variables of interest.We first included participants who had completed the 2011–2018 survey questionnaires. Individuals with missing age data, those younger than 45 years, or those lacking cumulative mean HbA1c/HDL-C data (2011–2015) were excluded, leaving 6,607 participants. We then excluded individuals with missing information on cardiometabolic multimorbidity (CMM), resulting in 5,175 participants. Finally, after removing cases with missing covariates such as gender or education, as well as those with extreme values of HbA1c/HDL-C, a total of 4,225 participants were included in the final analysis.

### 2.6. Statistical analysis

Continuous variables with a normal distribution were expressed as means and standard deviations (SD), while those with abnormal distribution were expressed as medians (interquartile range (IQR)). Categorical variables were expressed as frequencies and percentages (%). Baseline characteristics among groups, stratified by HbA1c/HDL-C quartiles (Q1, Q2, Q3, and Q4), were analyzed using the Kruskal-Wallis H test or Wilcoxon rank-sum test for continuous variables and the Chi-square test or Fisher’s exact test for categorical variables. The HbA1c/HDL-C ratio was analyzed as a continuous variable, with an increment of one IQR representing “HbA1c/HDL-C per IQR”. A multivariable logistic regression model was used to estimate the odds ratio (OR) and 95% confidence interval (CI) for CMM. A multivariate logistic regression model was established to determine the association between the cumulative average HbA1c/HDL-C (2011–2015) and the incidence of newly diagnosed CMM (2015–2018). Four models were applied to investigate the association between HbA1c/HDL-C and CMM: Model 1: unadjusted crude model. Model 2: adjusted for age, gender, education level, residence, marital status, and BMI. Model 3: further adjusted for smoking and drinking based on Model 2. Model 4: further adjusted for TC, TG, FBG, CRP, Hb, BUN, Cr, UA, antihypertensive medications, antidiabetic medications, and antidyslipidemic medications based on Model 3. Covariates included in the regression models were selected based on their associations with either HbA1c/HDL-C or CMM. For variables that demonstrated significance in the univariate analysis, a collinearity assessment was conducted using the variance inflation factor (VIF), where a VIF greater than five indicated significant collinearity [16]. All variables met the logistic regression analysis criteria, and no significant multicollinearity was observed (variance inflation factor for all variables <5) (Table S2 in S2 File). A multivariable-adjusted RCS was applied to evaluate the association between HbA1c/HDL-C and CMM risk, visually displaying the dose-response relationship between HbA1c/HDL-C levels and the incidence of CMM. The Akaike Information Criterion (AIC) and Bayesian Information Criterion (BIC) were used to determine the optimal number of knots, which was set to three. Subgroup analyses were conducted to examine potential interactions between sociodemographic characteristics, health-related behaviors, and BMI. A two-sided *p*-value < 0.05 was considered statistically significant. All statistical analyses were performed using RStudio v4.4.2.

## 3. Results

### 3.1. Baseline characteristics

In this study, a total of 4,225 participants were included, with a median baseline age of 58.17 years and female predominance (n = 2,360, 55.86%), and by the end of the follow-up period, 716 participants (16.95%) had developed CMM. The overall cumulative average HbA1c/HDL-C ratio of all participants from 2011 to 2015 was 4.38 (SD: 1.12). Table 1 details the baseline clinical characteristics across the quartiles of HbA1c/HDL-C, showing significant differences in gender, marital status, education level, residence, smoking behavior, and drinking status (*p* < 0. 05). Participants in the higher cumulative average HbA1c/HDL-C groups exhibited higher levels of FBG, TG, and Hb (*p* < 0. 05). There were no significant differences in age or BMI among the groups. A comparison of the overall characteristics between individuals with and without CMM is shown in S3 Table in S2 File.

**Table 1:** Baseline characteristics of participants (n = 4,225).

Characteristic	Q1(1.64–3.59)	Q2(3.59–4.23)	Q3(4.23–5.00)	Q4(5.00–8.98)	*p* value
Age	58.44 ± 8.53	58.12 ± 8.89	57.82 ± 8.32	58.30 ± 8.41	**0.361**
Gender					**<0.001**
Female	673(58.88)	672(60.00)	592(55.28)	423(47.47)	
Male	470(41.12)	448(40.00)	479(44.72)	468(52.53)	
Marital					**0.029**
Married	1027(89.85)	988(88.21)	977(91.22)	818(91.81)	
Non-married	116(10.15)	132(11.79)	94(8.78)	73(8.19)	
Education					**0.017**
College or above	22(1.92)	27(2.41)	25(2.33)	23(2.58)	
High school	270(23.62)	274(24.46)	292(27.26)	269(30.19)	
Primary school or below	851(74.45)	819(73.12)	754(70.40)	599(67.23)	
Residence					**0.007**
City/town	132(11.55)	123(11.98)	147(13.73)	139(15.60)	
Village	1011(88.45)	997(89.02)	924(86.27)	752(84.40)	
Smoking					**<0.001**
Current smoker	338(29.57)	301(26.88)	325(30.35)	297(33.33)	
Ex-smoker	70(6.12)	76(6.79)	80(7.47)	85(9.54)	
Non-smoker	735(64.30)	743(66.34)	666(62.18)	509(57.13)	
Drinking					**<0.001**
Drink but less than once a month	87(7.61)	94(8.39)	93(8.68)	75(8.42)	
Drink more than once a month	327(28.61)	208(18.57)	200(18.67)	181(20.31)	
None of these	729(63.78)	818(73.04)	778(72.64)	635(71.27)	
Antihypertensive medications	112 (9.80)	125 (11.16)	151 (14.10)	165(18.52)	**<0.001**
Antidiabetic medications	5 (0.44).	8 (0.71)	9 (0.84).	40 (4.49)	**<0.001**
Antidyslipidemic medications	23 (2.01)	36 (3.21)	38 (3.55)	64 (7.18)	**<0.001**
BUN(mg/dL)	16.19 ± 4.64	15.45 ± 4.27	15.31 ± 4.19	15.39 ± 4.06	**<0.001**
FBG (mg/dL)	100.91 ± 15.58	103.30 ± 19.52	104.65 ± 19.06	117.50 ± 42.42	**<0.001**
Cr(mg/dL)	0.75 ± 0.16	0.75 ± 0.19	0.76 ± 0.17	0.78 ± 0.18	**<0.001**
TC(mg/dL)	199.63 ± 34.65	192.57 ± 37.11	189.12 ± 36.58	188.26 ± 39.36	**<0.001**
TG (mg/dL)	89.45 ± 43.72	109.81 ± 56.36	130.69 ± 71.68	190.40 ± 129.52	**<0.001**
HDL-C (mg/dL)	33.34 ± 4.97	26.61 ± 2.18	22.82 ± 1.94	19.22 ± 2.92	**<0.001**
LDL-C (mg/dL)	115.44 ± 31.52	119.11 ± 33.56	118.65 ± 32.96	110.76 ± 37.06	**<0.001**
CRP(mg/dL)	1.76 ± 5.60	2.46 ± 8.11	2.28 ± 5.15	3.16 ± 8.77	**<0.001**
Hb(g/dL)	14.01 ± 2.10	14.39 ± 2.20	14.39 ± 2.30	14.66 ± 2.25	**<0.001**
HbA1c(%)	2.66 ± 0.17	2.73 ± 0.18	2.76 ± 0.19	2.96 ± 0.49	**<0.001**
UA (mg/dL)	4.19 ± 1.15	4.18 ± 1.13	4.33 ± 1.18	4.57 ± 1.23	**<0.001**
BMI (kg/m²)	21.87 ± 3.23	25.75 ± 73.94	24.36 ± 14.44	24.93 ± 3.73	**0.102**
HbA1c/HDL-C(mmol/L)	3.15 ± 0.36	3.99 ± 0.20	4.71 ± 0.24	6.06 ± 0.80	**<0.001**

### 3.2. The relationship between HbA1c/HDL-C and the prevalence of CMM in the Chinese middle-aged and elderly population

**Fig 2:**
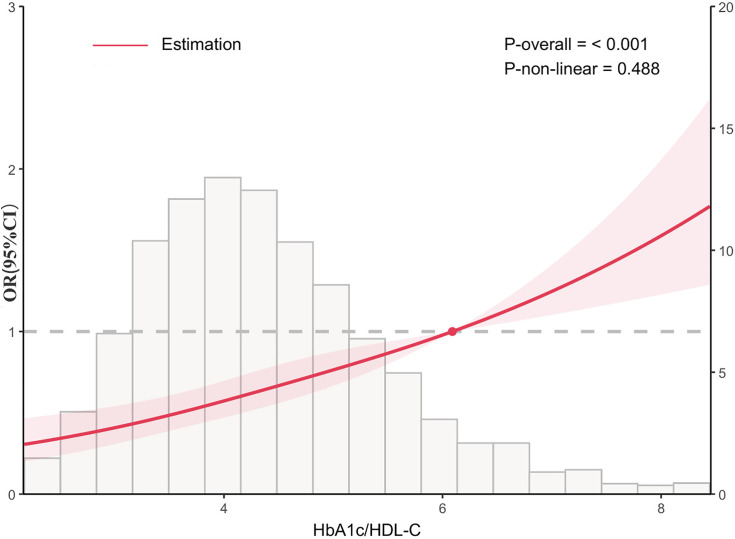
Restricted cubic spline of HbA1c/HDL-C and CMM risk. The model was adjusted for age, gender, marital status, educational level, residence, smoking status, drinking status, BUN, FBG, Cr, TC, TG, CRP, Hb, HbA1c, UA, BMI, antihypertensive medications, antidiabetic medications, and antidyslipidemic medications. Odds ratios were represented by red solid lines, and their corresponding 95% confidence intervals by red shaded areas.

We next conducted multivariable logistic regression based on the quartiles of the cumulative average HbA1c/HDL-C. Table 2 presents the association between HbA1c/HDL-C and the risk of CMM, as well as the quartiles of HbA1c/HDL-C. HbA1c/HDL-C, as a continuous variable, remained significantly associated with the risk of CMM after adjusting for different covariates (Model 1: OR = 1.07, 95% CI = 1.05, 1.09; Model 2: OR = 1.07, 95% CI = 1.06, 1.09; Model 3: OR = 1.07, 95% CI = 1.06, 1.09); Model 4: OR = 1.05, 95% CI = 1.04, 1.08). In all models, each IQR increase in HbA1c/HDL-C significantly increased the risk of CMM, and this association remained consistent after multivariable adjustment. Compared to the first quartile of HbA1c/HDL-C (Q1), Q4 exhibited the highest risk of CMM in Model 4, which was adjusted for age, gender, marital status, education level, residence, smoking status, drinking status, BUN, FBG, Cr, TC, TG, CRP, Hb, HbA1c, UA, BMI, antihypertensive medications, antidiabetic medications, and antidyslipidemic medications(OR = 1.08, 95% CI = 1.04, 1.12). Furthermore, the relationship between the cumulative average HbA1c/HDL-C ratio and CMM was investigated using multivariable-adjusted RCS analysis. A positive association was found between HbA1c/HDL-C and the incidence of CMM. The results showed that, after adjusting for all potential confounders, there was a linear relationship between cumulative average HbA1c/HDL-C and the risk of CMM (*p*-nonlinear = 0.547). The cumulative risk of CMM was observed to gradually increase when the cumulative average HbA1c/HDL-C ratio was ≥ 6.453, while a lower risk of CMM was noted when this ratio was < 6.453 (Fig 2).

**Table 2:** Association between HbA1c/HDL-C and the risk of CMM.

Cumulative mean HbA1c/HDL-C	New stroke incidence	
	OR (95% CI)	*p* value
**Model 1.**		
**HbA1c/HDL-C per IQR**	1.07(1.05,1.09)	<0.001
**Quartiles of HbA1c/HDL-C**		
Q1	Ref	
Q2	1.01(0.98,1.05)	0.394
Q3	1.05(1.02,1.08)	0.002
Q4	1.13(1.09,1.16)	<0.001
**Model 2**		
**HbA1c/HDL-C per IQR**	1.07(1.06,1.09)	<0.001
**Quartiles of HbA1c/HDL-C**		
Q1	Ref	
Q2	1.01(0.98,1.05)	0.371
Q3	1.06(1.02,1.09)	<0.001
Q4	1.13(1.10,1.17)	<0.001
**Model 3**		
**HbA1c/HDL-C per IQR**	1.07(1.06,1.09)	<0.001
**Quartiles of HbA1c/HDL-C**		
Q1	Ref	
Q2	1.01(0.98,1.04)	0.431
Q3	1.05(1.02,1.09)	<0.001
Q4	1.13(1.09,1.17)	<0.001
**Model 4**		
**HbA1c/HDL-C per IQR**	1.05(1.04,1.08)	<0.001
**Quartiles of HbA1c/HDL-C**		
Q1	Ref	
Q2	1.00(0.97,1.04)	0.763
Q3	1.04(1.01,1.07)	0.017
Q4	1.08(1.04,1.12)	<0.001

### 3.3. Stratified analysis

**Fig 3:**
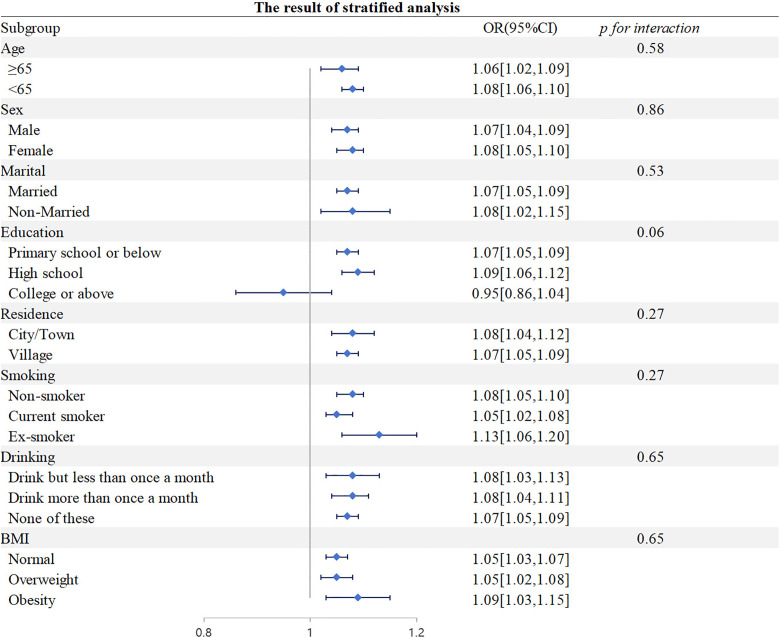
Stratified analysis.

This study also accounted for factors such as age, gender, marital status, education level, residence, smoking status, drinking status, and BMI, grouping participants for stratified analysis. The association between HbA1c/HDL-C and CMM incidence was present in all subgroups except for the education level subgroup. Stratified analysis showed no interaction between HbA1c/HDL-C and factors such as age, gender, marital status, education level, residence, smoking status, drinking status, and BMI (Fig 3).

## 4. Discussion

The HbA1c/HDL-C ratio, a composite index integrating the glycometabolic disorder indicator HbA1c and the lipid metabolic disorder indicator HDL-C, has attracted widespread interest from researchers. Previous studies have reported that a higher HbA1c/HDL-C ratio is associated with an increased risk of stroke in the middle-aged and elderly Chinese population, demonstrating a robust positive linear relationship with stroke risk [17]. To our knowledge, no studies have hitherto assessed the association between HbA1c/HDL-C and the risk of CMM. Our study revealed a linear relationship between HbA1c/HDL-C and CMM risk, indicating that a higher HbA1c/HDL-C value is associated with a greater risk of CMM. This finding provides a new perspective for predicting CMM.

Based on the baseline data from the 2011 CHARLS database, we found that participants who developed CMM were typically older, female, married, rural residents with lower education levels. They also exhibited higher levels of BUN, FBG, TC, TG, LDL, CRP, hemoglobin, HbA1c, UA, and BMI, while having lower LDL-C levels. Compared to non-CMM participants, those with CMM exhibited significantly higher HbA1c/HDL-C levels. In the fully adjusted model, the highest risk of CMM was observed in the fourth quartile of HbA1c/HDL-C (Q4) (OR = 1.08, 95% CI: 1.04–1.12). An OR of 1.08 indicates that for each one-unit increase in HbA1c/HDL-C, the risk of developing CMM increases by only 8%. However, considering the high prevalence of CMM in the middle-aged and elderly population, even a modest increase in risk may translate into a substantial public health burden at the population level. Moreover, since the HbA1c/HDL-C ratio can be derived from routine medical check-up data, it offers a practical and accessible way to identify individuals at risk early, highlighting its potential value in public health. Consistently, small effect sizes have been reported in cardiovascular epidemiology studies. In this respect, several variables in the Framingham Risk Score demonstrate similarly modest but cumulatively significant impacts on cardiovascular risk [18]. The narrow confidence interval for the OR (span of 0.08) suggests a robust estimate, indicative of a large sample size or low variability in the data, thereby lending stability and reliability to the OR estimate. Analysis of the follow-up data revealed a positive association between HbA1c/HDL-C and the risk of CMM. After fully adjusting for confounding factors, each IQR increase in HbA1c/HDL-C was associated with a 5% higher risk of developing CMM (OR=1.05, 95% CI: 1.04–1.08). In the subsequent dose-response analysis, a linear relationship was found between HbA1c/HDL-C and CMM risk. Specifically, while the risk of CMM remained low in the lower range, an increase in HbA1c/HDL-C beyond a certain threshold was positively correlated with a higher CMM risk. Stratified analyses across subgroups further supported these findings.

Among comorbidities, cardiometabolic comorbidity is one of the most extensively studied patterns [5], defined as the coexistence of at least two cardiometabolic diseases, including diabetes, stroke, and heart disease [6]. The composite index HbA1c/HDL-C is particularly relevant since both HbA1c and HDL-C are closely associated with diabetes, stroke, and heart disease. In 2010, the American Diabetes Association incorporated HbA1c ≥ 6.5% into the revised diagnostic criteria for diabetes [19]. Besides, a large meta-analysis indicated that elevated HbA1c levels are associated with an increased risk of first stroke in diabetic patients [20]. Cohort studies have also reported that HbA1c levels are associated with the prognosis of patients with coronary heart disease and that HbA1c concentration can be used to predict cardiovascular disease risk, helping to identify high-risk individuals for targeted interventions [21,22]. HDL particles have been established to harbor anti-atherosclerotic properties [23]. In this respect, the Framingham Study reported an inverse relationship between HDL-C levels and the risk of coronary heart disease, suggesting that therapeutically increasing HDL could help prevent coronary heart disease [24]. A study based on the UK Biobank found that HDL may lose its atheroprotective function in diabetic patients [25], indicating a unique interaction between HDL and diabetes. Similarly, HDL-C levels have been found to have an inverse relationship with stroke risk [26,27]. Although the relationships between HbA1c, HDL-C, and diabetes, stroke, and heart disease have been widely discussed, the role of HbA1c/HDL-C in multimorbidity remains insufficiently explored. Our study found a robust linear relationship between HbA1c/HDL-C and the risk of CMM, indicating that higher ratios are associated with an increased likelihood of CMM. This elevation may indicate an increase in chronic inflammation, insulin resistance, and the risk of metabolic syndrome. The underlying biological mechanisms can be explained from several perspectives. First, an excessively elevated HbA1c/HDL-C ratio is a hallmark of metabolic imbalance. The adverse metabolic state characterized by high HbA1c, accompanied by low HDL-C, reflects disorders in glucose and lipid metabolism. High HbA1c indicates poor glycemic control, and a hyperglycemic environment reduces endothelial nitric oxide (NO) production, leading to impaired vasodilation, increased vascular resistance, endothelial dysfunction, and chronic low-grade inflammation [28–31]. Meanwhile, low HDL-C weakens its anti-atherosclerotic effects, allowing cholesterol to accumulate in the arterial walls and accelerating atherosclerosis [32]. Besides, inflammation and oxidative stress exacerbate cardiometabolic risk [33,34]. A hyperglycemic environment promotes the release of pro-inflammatory cytokines such as IL-6 and TNF-α, leading to systemic chronic inflammation. This inflammatory response aggravates insulin resistance, impairs HDL-C’s protective functions, and increases the risk of CMM [35]. Furthermore, high blood glucose levels increase oxidative stress, leading to the oxidation of LDL-C and the formation of oxidized LDL (ox-LDL), which in turn promotes the development of atherosclerosis. Low HDL-C levels fail to effectively clear oxidative byproducts, worsening vascular damage [36,37]. Besides, insulin resistance contributes to lipid metabolism disorders. A hyperinsulinemic state inhibits lipolysis while promoting the increase of very-low-density lipoprotein (VLDL) and LDL, along with a decrease in HDL-C, thereby accelerating the progression of atherosclerosis [38]. An elevated HbA1c/HDL-C ratio indicates dual abnormalities in glucose and lipid metabolism. These metabolic disorders accumulate through multiple pathways, leading to atherosclerosis, cardiovascular diseases, and type 2 diabetes. A lower HbA1c/HDL-C level helps maintain metabolic balance and reduces the risk of CMM, whereas a higher HbA1c/HDL-C level signifies metabolic dysfunction, increased inflammatory responses, and greater vascular damage, thereby increasing CMM risk. Overall, our study suggests that by combining the biomarkers HbA1c and HDL-C, the HbA1c/HDL-C ratio provides a more comprehensive reflection of the relationship between dysregulated glucose-lipid metabolism and cardiometabolic diseases.

This study presents several notable strengths in its research design and methodology. First, it introduces the composite glucose-lipid metabolic index, HbA1c/HDL-C, as a novel predictor of CMM risk, providing a quantitative approach to assess the combined effects of glucose and lipid metabolism, which is of significant innovation. Second, our longitudinal analysis utilized cumulative average parameters rather than single time-point measurements, thereby facilitating a more accurate representation of long-term glucose-lipid metabolism. Finally, the study adjusted for multiple confounding factors, significantly reducing bias, and conducted subgroup analyses based on age, gender, and other factors to further validate the robustness of the findings. However, this study also has several limitations that should be acknowledged. First, CMM was diagnosed based on self-reported physician assessments, which may introduce potential information bias. Future large-scale randomized controlled trials are needed to confirm our findings. Second, although the study adjusted for multiple confounding factors (such as age, gender, smoking, drinking, and medication use), there may still be unmeasured confounders, including dietary intake, physical activity levels, genetic factors, and environmental exposures. These may lead to residual confounding and affect the interpretation of the results. Third, since a large amount of missing data was not completely random, the final sample may not fully represent the Chinese middle-aged and elderly population, potentially introducing selection bias and limiting the generalizability of our findings. These factors should be taken into consideration in future research.

## 5. Conclusion

This study demonstrates a significant linear relationship between the HbA1c/HDL-C ratio and the risk of developing CMM. The HbA1c/HDL-C ratio may be a potential independent risk marker for CMM. Therefore, among middle-aged and older adults, monitoring HbA1c/HDL-C levels may help identify individuals at high risk of developing CMM.

## Supporting information

S1 File Sampling strategy of the survey. (PDF)

S2 File Table1. Distribution of variables with missing data. **Table2.** Diagnostic steps for collinearity between HbA1C/HDL-C and other covariates.**Table3.** Baseline characteristics of the study participants.
(DOCX)

## References

[1] Xu X, Mishra GD, Dobson AJ, Jones M. Progression of diabetes, heart disease, and stroke multimorbidity in middle-aged women: A 20-year cohort study. PLoS Med. 2018;15(3):e1002516. doi: 10.1371/journal.pmed.1002516 29534066 PMC5849280

[2] Martín-Timón I, Sevillano-Collantes C, Segura-Galindo A, Del Cañizo-Gómez FJ. Type 2 diabetes and cardiovascular disease: Have all risk factors the same strength?. World J Diabetes. 2014;5(4):444–70. doi: 10.4239/wjd.v5.i4.444 25126392 PMC4127581

[3] Skou ST, Mair FS, Fortin M, Guthrie B, Nunes BP, Miranda JJ, et al. Multimorbidity. Nat Rev Dis Primers. 2022;8(1):48. doi: 10.1038/s41572-022-00376-4 35835758 PMC7613517

[4] Langenberg C, Hingorani AD, Whitty CJM. Biological and functional multimorbidity-from mechanisms to management. Nat Med. 2023;29(7):1649–57. doi: 10.1038/s41591-023-02420-6 37464031

[5] Busija L, Lim K, Szoeke C, Sanders KM, McCabe MP. Do replicable profiles of multimorbidity exist? Systematic review and synthesis. Eur J Epidemiol. 2019;34(11):1025–53. doi: 10.1007/s10654-019-00568-5 31624969

[6] Han Y, Hu Y, Yu C, Guo Y, Pei P, Yang L, et al. Lifestyle, cardiometabolic disease, and multimorbidity in a prospective Chinese study. Eur Heart J. 2021;42(34):3374–84. doi: 10.1093/eurheartj/ehab413 34333624 PMC8423468

[7] Emerging Risk Factors Collaboration, Di Angelantonio E, Kaptoge S, Wormser D, Willeit P, Butterworth AS, et al. Association of Cardiometabolic Multimorbidity With Mortality. JAMA. 2015;314(1):52–60. doi: 10.1001/jama.2015.7008 26151266 PMC4664176

[8] Otero YF, Stafford JM, McGuinness OP. Pathway-selective insulin resistance and metabolic disease: the importance of nutrient flux. J Biol Chem. 2014;289(30):20462–9. doi: 10.1074/jbc.R114.576355 24907277 PMC4110258

[9] Hu X, Li W, Wang C, Zhang H, Lu H, Li G, et al. Association between the Plasma-Glycosylated Hemoglobin A1c/High-Density Lipoprotein Cholesterol Ratio and Carotid Atherosclerosis: A Retrospective Study. J Diabetes Res. 2021;2021:9238566. doi: 10.1155/2021/9238566 34805413 PMC8598339

[10] Huang C, You H, Zhang Y, Fan L, Feng X, Shao N. Association between the hemoglobin A1c/High-density lipoprotein cholesterol ratio and stroke incidence: a prospective nationwide cohort study in China. Lipids Health Dis. 2025;24(1):25. doi: 10.1186/s12944-025-02438-4 39863906 PMC11762894

[11] He S, Lu S, Yu C, Kuang M, Qiu J, Sheng G, et al. The newly proposed plasma-glycosylated hemoglobin A1c/High-Density lipoprotein cholesterol ratio serves as a simple and practical indicator for screening metabolic associated fatty liver disease: an observational study based on a physical examination population. BMC Gastroenterol. 2024;24(1):274. doi: 10.1186/s12876-024-03362-0 39160462 PMC11331873

[12] Zhao Y, Hu Y, Smith JP, Strauss J, Yang G. Cohort profile: the China Health and Retirement Longitudinal Study (CHARLS). Int J Epidemiol. 2014;43(1):61–8. doi: 10.1093/ije/dys203 23243115 PMC3937970

[13] American Diabetes Association Professional Practice Committee. 2. Classification and Diagnosis of Diabetes: Standards of Medical Care in Diabetes-2022. Diabetes Care. 2022;45(Suppl 1):S17–38. doi: 10.2337/dc22-S002 34964875

[14] Min Q, Wu Z, Yao J, Wang S, Duan L, Liu S, et al. Association between atherogenic index of plasma control level and incident cardiovascular disease in middle-aged and elderly Chinese individuals with abnormal glucose metabolism. Cardiovasc Diabetol. 2024;23(1):54. doi: 10.1186/s12933-024-02144-y 38331798 PMC10854096

[15] Xiong C-C, Gao F, Zhang J-H, Ruan Y, Gao T-G, Cai J-Y, et al. Investigating the impact of remnant cholesterol on new-onset stroke across diverse inflammation levels: Insights from the China Health and Retirement Longitudinal Study (CHARLS). Int J Cardiol. 2024;405:131946. doi: 10.1016/j.ijcard.2024.131946 38460732

[16] Peng L, Liu Y, Wu W, Zhou Y, Huang X. Unhealthy diet and lifestyle factors linked to female androgenetic alopecia: a community-based study from Jidong study, China. BMC Public Health. 2025;25(1):606. doi: 10.1186/s12889-025-21560-7 39948511 PMC11827360

[17] Xu M, Zhang L, Xu D, Shi W, Zhang W. Usefulness of C-reactive protein-triglyceride glucose index in detecting prevalent coronary heart disease: findings from the National Health and Nutrition Examination Survey 1999-2018. Front Cardiovasc Med. 2024;11:1485538. doi: 10.3389/fcvm.2024.1485538 39473894 PMC11518723

[18] D’Agostino RB Sr, Vasan RS, Pencina MJ, Wolf PA, Cobain M, Massaro JM, et al. General cardiovascular risk profile for use in primary care: the Framingham Heart Study. Circulation. 2008;117(6):743–53. doi: 10.1161/CIRCULATIONAHA.107.699579 18212285

[19] Ding L, Xu Y, Liu S, Bi Y, Xu Y. Hemoglobin A1c and diagnosis of diabetes. J Diabetes. 2018;10(5):365–72. doi: 10.1111/1753-0407.12640 29292842

[20] Mitsios JP, Ekinci EI, Mitsios GP, Churilov L, Thijs V. Relationship Between Glycated Hemoglobin and Stroke Risk: A Systematic Review and Meta-Analysis. J Am Heart Assoc. 2018;7(11):e007858. doi: 10.1161/JAHA.117.007858 29773578 PMC6015363

[21] Li Q, Yuan D, Zeng G, Jiang L, Xu L, Xu J, et al. The association between glycated hemoglobin levels and long-term prognosis in patients with diabetes and triple-vessel coronary disease across different age groups: A cohort study. Diabetes Res Clin Pract. 2024;213:111751. doi: 10.1016/j.diabres.2024.111751 38906334

[22] Khaw K-T, Wareham N. Glycated hemoglobin as a marker of cardiovascular risk. Curr Opin Lipidol. 2006;17(6):637–43. doi: 10.1097/MOL.0b013e3280106b95 17095908

[23] Chapman MJ. Therapeutic elevation of HDL-cholesterol to prevent atherosclerosis and coronary heart disease. Pharmacol Ther. 2006;111(3):893–908. doi: 10.1016/j.pharmthera.2006.02.003 16574234

[24] Castelli WP, Garrison RJ, Wilson PW, Abbott RD, Kalousdian S, Kannel WB. Incidence of coronary heart disease and lipoprotein cholesterol levels. The Framingham Study. JAMA. 1986;256(20):2835–8. doi: 10.1001/jama.1986.03380200073024 3773200

[25] Chen J-X, Lu Q, Geng T, Wang Y, Wang Y, Li R, et al. Differences in HDL-related coronary heart disease risk between individuals with and without diabetes. Atherosclerosis. 2024;397:118553. doi: 10.1016/j.atherosclerosis.2024.118553 39186911

[26] Amarenco P, Labreuche J, Touboul P-J. High-density lipoprotein-cholesterol and risk of stroke and carotid atherosclerosis: a systematic review. Atherosclerosis. 2008;196(2):489–96. doi: 10.1016/j.atherosclerosis.2007.07.033 17923134

[27] Hu Y, Song M, Wu D, Zhang Y, Li G, Luo H. The association between HDL-C and stroke in the middle-aged and elderly: A cross-sectional study. Brain Behav. 2023;13(3):e2901. doi: 10.1002/brb3.2901 36749609 PMC10013934

[28] Shi Y, Vanhoutte PM. Macro- and microvascular endothelial dysfunction in diabetes. J Diabetes. 2017;9(5):434–49. doi: 10.1111/1753-0407.12521 28044409

[29] Sharma C, Suliman A, Al Hamad SM, Yasin J, Abuzakouk M, AlKaabi J, et al. Association of Biomarkers for Dyslipidemia, Inflammation, and Oxidative Stress with Endothelial Dysfunction in Obese Youths: A Case-Control Study. Diabetes Metab Syndr Obes. 2024;17:2533–45. doi: 10.2147/DMSO.S458233 38915900 PMC11194285

[30] Škrha J, Šoupal J, Škrha J Jr, Prázný M. Glucose variability, HbA1c and microvascular complications. Rev Endocr Metab Disord. 2016;17(1):103–10. doi: 10.1007/s11154-016-9347-2 26975588

[31] Sun D, van Greevenbroek MMJ, Scheijen JLJM, Kelly J, Schalkwijk CG, Wouters K. Methylglyoxal Mediates the Association Between 2-Hour Plasma Glucose and HbA1c With Inflammation: The Maastricht Study. J Clin Endocrinol Metab. 2025;110(7):2047–54. doi: 10.1210/clinem/dgae640 39315630 PMC12187126

[32] Adorni MP, Ronda N, Bernini F, Zimetti F. High Density Lipoprotein Cholesterol Efflux Capacity and Atherosclerosis in Cardiovascular Disease: Pathophysiological Aspects and Pharmacological Perspectives. Cells. 2021;10(3):574. doi: 10.3390/cells10030574 33807918 PMC8002038

[33] Nishida K, Otsu K. Inflammation and metabolic cardiomyopathy. Cardiovasc Res. 2017;113(4):389–98. doi: 10.1093/cvr/cvx012 28395010

[34] Chen Z, Jin Z-X, Cai J, Li R, Deng K-Q, Ji Y-X, et al. Energy substrate metabolism and oxidative stress in metabolic cardiomyopathy. J Mol Med (Berl). 2022;100(12):1721–39. doi: 10.1007/s00109-022-02269-1 36396746

[35] Silveira Rossi JL, Barbalho SM, Reverete de Araujo R, Bechara MD, Sloan KP, Sloan LA. Metabolic syndrome and cardiovascular diseases: Going beyond traditional risk factors. Diabetes Metab Res Rev. 2022;38(3):e3502. doi: 10.1002/dmrr.3502 34614543

[36] Gardner CD, Fortmann SP, Krauss RM. Association of small low-density lipoprotein particles with the incidence of coronary artery disease in men and women. JAMA. 1996;276(11):875–81. doi: 10.1001/jama.1996.03540110029028 8782636

[37] Taskinen M-R. Type 2 diabetes as a lipid disorder. Curr Mol Med. 2005;5(3):297–308. doi: 10.2174/1566524053766086 15892649

[38] Luciani L, Pedrelli M, Parini P. Modification of lipoprotein metabolism and function driving atherogenesis in diabetes. Atherosclerosis. 2024;394:117545. doi: 10.1016/j.atherosclerosis.2024.117545 38688749

