# Supplementary material for: The association between HbA1c/HDL-C and the incidence of cardiometabolic multimorbidity in middle-aged and elderly adults: Results from the China Health and Retirement Longitudinal Study
Source: PLoS One. 2025 Oct 23;20(10):e0332376. doi: 10.1371/journal.pone.0332376 (PMC12548882; doi:10.1371/journal.pone.0332376)
Supplement: S1 File — (PDF) [file pone.0332376.s001.pdf]

S1 File.

Sampling strategy of the survey

The CHARLS, following the design of the Health and Retirement Study (HRS), The English Longitudinal Study of Ageing (ELSA), The Survey of Health, Ageing and Retirement in Europe (SHARE), and other renowned ageing surveys, collects a nationally representative sample of Chinese residents aged 45 and over dedicated to inform scientific research on the elderly and assess their health trends[1]. Its baseline survey covered 450 villages and communities across the country, with a sample size of 17,708 individuals in 10,257 households. The CHARLS uses a strategy of multi-stage systematic random sampling to select respondents. First, district and county units are implicitly stratified by region, rural or urban areas, and GDP per capita. Based on PPS (Probability Proportional to population Size), 150 district and county units were randomly chosen among all county units, and three village or community units (psus) were further randomly selected within each county unit. The team developed a special mapping software to help draw a sample frame of all households in each psu. Within each household selected from the mapping frame, one resident 45 and over was randomly chosen to be the main respondent, and this person's spouse was automatically included in the sample. To avoid human error and manipulation, each sampling stage was computerized, and all interviews were conducted using computer-aided personal interview (CAPI) technology. Thanks to the strict quality control implemented in the sampling and survey process, the CHARLS sample is representative of the population of older adults in the country. Each individual is initially assigned a sampling weight, for which a logit model is used to correct the nonresponse bias. The post-hoc weights applied in this study are individual weights further adjusted according to the demographic distributions from the population census and the population sample survey conducted by the National Bureau of Statistics.

1. Zhao Y, Hu Y, Smith JP, Strauss J, Yang G. Cohort profile: the China Health and Retirement Longitudinal Study (CHARLS). Int J Epidemiol. 2014;43(1):61-8. Epub 2012/12/18. doi: 10.1093/ije/dys203. PubMed PMID: 23243115; PubMed Central PMCID: PMCPMC3937970.
