# Supplementary material for: The association between HbA1c/HDL-C and the incidence of cardiometabolic multimorbidity in middle-aged and elderly adults: Results from the China Health and Retirement Longitudinal Study
Source: PLoS One. 2025 Oct 23;20(10):e0332376. doi: 10.1371/journal.pone.0332376 (PMC12548882; doi:10.1371/journal.pone.0332376)
Supplement: S2 File — Table2. Diagnostic steps for collinearity between HbA1C/HDL-C and other covariates.Table3. Baseline characteristics of the study participants. (DOCX) [file pone.0332376.s002.docx]

| Variables | Number of Missing | Missing proportion |
| --- | --- | --- |
| BMI | 579 | 11.188406 |
| Drinking status | 289 | 5.584541 |
| Hemoglobin | 90 | 1.739130 |
| Smoking status | 224 | 1.333333 |
| Creatinine | 13 | 0.251208 |
| FBG | 12 | 0.231884 |
| LDL | 10 | 0.193237 |
| TC | 7 | 0.135266 |
| TG | 5 | 0.096618 |
| Residence | 2 | 0.038647 |
| UA | 1 | 0.019324 |
| CRP | 1 | 0.019324 |
| BUN | 1 | 0.019324 |

**S2 Table.** Distribution of variables with missing data.

**S2 Table2.** Diagnostic steps for collinearity between HbA1C/HDL-C and other covariates.

| Variables | GVIF | Df | GVIF^^(1/2Df)^ |
| --- | --- | --- | --- |
| HbA1c/HDL-C | 1.533970 | 1 | 1.238535 |
| Age | 1.314589 | 1 | 1.146555 |
| Gender | 2.984670 | 1 | 1.727620 |
| Marital status | 1.100414 | 1 | 1.049006 |
| Education levels | 1.341807 | 2 | 1.076273 |
| Residence | 1.155055 | 1 | 1.074735 |
| Smoking status | 2.106383 | 2 | 1.204715 |
| Drinking status | 1.440728 | 2 | 1.095583 |
| Antihypertensive medications | 1.063081 | 1 | 1.031058 |
| Antidiabetic medications | 1.176551 | 1 | 1.084689 |
| Antidyslipidemic medications | 1.067620 | 1 | 1.033257 |
| BUN | 1.141588 | 1 | 1.068451 |
| FBG | 1.258906 | 1 | 1.122010 |
| Cr | 1.748339 | 1 | 1.322248 |
| Hb | 1.177190 | 1 | 1.084984 |
| TG | 1.515626 | 1 | 1.231108 |
| CRP | 1.015605 | 1 | 1.007772 |
| UA | 1.537782 | 1 | 1.240074 |
| BMI | 1.008077 | 1 | 1.004030 |

VIF = 1/(1-R^2^). The variables with VIF>5 will be regarded as collinear variables and cannot be included in the multiple regression model.

**Notes:** HbA1c, hemoglobin A1c; HDL-C, high-density lipoprotein cholesterol; BUN,blood urea nitrogen; FBG, fasting blood glucose;Cr,serum creatinine; Hb,hemoglobin; TG, triglyceride;CRP,C-reactive protein;UA, uric acid;BMI, body mass index;VIF,variance inflation factor

**S3 Table3.**Baseline characteristics of the study participants.

| Characteristic | Total (n=4,225) | CMM | | P value |
| --- | --- | --- | --- | --- |
|  |  | No  (n=n=3,509) | Yes  (n=716) |  |
| Age, year | 58.17±8.55 | 57.74±8.52 | 60.27±8.38 | **<0.001** |
| Gender |  |  |  | **0.199** |
| Female | 2360(55.86) | 1944(55.40) | 416(58.10) |  |
| Male | 1865(44.14) | 1565(44.60) | 300(41.90) |  |
| Marital |  |  |  | **0.002** |
| Married | 3810(90.18) | 3187(90.82) | 623(87.01) |  |
| Non-Married | 415(9.82) | 322(9.18) | 93(12.99) |  |
| Education |  |  |  | **0.031** |
| College or above | 97 (2.30) | 80(2.28) | 17(2.37) |  |
| High school | 1105(26.15) | 946(26.96) | 159(22.21) |  |
| Primary school or below | 3023(71.55) | 2483(70.76) | 540(75.42) |  |
| Residence |  |  |  | **0.525** |
| City/town | 541(12.80) | 455(12.97) | 86(12.01) |  |
| Village | 3684(87.20) | 3054(87.03) | 630(87.99) |  |
| Smoking |  |  |  | **<0.001** |
| Current smoker | 1261(29.85) | 1082(30.83) | 179(25.00) |  |
| Ex-smoker | 311(7.36) | 233(6.64) | 78(10.89) |  |
| Non-smoker | 2653(62.79) | 2194(62.52) | 459(64.11) |  |
| Drinking |  |  |  | **0.127** |
| Drink but less than once a month | 349(8.26) | 298(8.49) | 51(7.12) |  |
| Drink more than once a month | 916(21.68) | 775(22.09) | 141(19.69) |  |
| None of these | 2960(70.06) | 2436(69.42) | 524(73.18) | **0.794** |
| Antihypertensive medications | 553(13.09) | 341(9.72) | 212(29.61) | **<0.001** |
| Antidiabetic medications | 62(1.47) | 33(0.94) | 29(4.05) | **<0.001** |
| Antidyslipidemic medications | 161(3.81) | 103(2.94) | 58(8.10) | **<0.001** |
| BUN(mg/dL) | 15.60±4.33 | 15.61±4.31 | 15.56±4.40 | **<0.001** |
| FBG (mg/dL) | 105.99±25.98 | 104.55±22.44 | 113.07±38.17 | **<0.001** |
| Cr(mg/dL) | 0.76±0.18 | 0.76±0.18 | 0.77±0.18 | **0.405** |
| TC(mg/dL) | 192.69±37.08 | 192.03±36.97 | 195.96±37.51 | **<0.001** |
| TG (mg/dL) | 126.59±86.63 | 124.13±85.37 | 138.63±91.72 | **<0.001** |
| HDL-C (mg/dL) | 25.91±6.13 | 26.10±6.19 | 25.01±5.75 | **<0.001** |
| LDL-C (mg/dL) | 116.24±33.79 | 115.68±33.36 | 118.99±35.74 | **0.017** |
| CRP(mg/dL) | 2.37±7.00 | 2.29±7.05 | 2.77±6.75 | **0.095** |
| Hb(g/dL) | 14.35±2.22 | 14.33±2.22 | 14.44±2.23 | **0.224** |
| HbA1c(%) | 2.77±0.30 | 2.74±0.25 | 2.89±0.44 | **<0.001** |
| UA (mg/dL) | 4.30±1.18 | 4.29±1.17 | 4.38±1.21 | **0.073** |
| HbA1c/HDL-C(mmol/L) | 4.38±1.12 | 4.31±1.09 | 4.71±1.23 | **<0.001** |

**Notes:** BUN,blood urea nitrogen; FBG, fasting blood glucose;Cr,serum creatinine;TC,total cholesterol;TG,triglycerides;HDL-C,high-density lipoprotein cholesterol;LDL-C,low density lipoprotein cholesterol;CRP,C-reactive protein; Hb,hemoglobin; HbA1c, glycosylated hemoglobin A1c;UA, uric acid
